# 4-Amino-3,5-dimethyl-4*H*-1,2,4-triazole

**DOI:** 10.1107/S1600536808014815

**Published:** 2008-05-24

**Authors:** Daojin Li, Guo-Chang Wei, Shu-Zhi Song, Hui Wang

**Affiliations:** a College of Chemistry and Chemical Engineering, Luoyang Normal University, Luoyang 471022, People’s Republic of China; b Hebei Zhongrun Pharmaceutical Co. Ltd, Shijiazhuang Pharm Group Co. Ltd, Shijiazhuang 050041, People’s Republic of China

## Abstract

In the title compound, C_4_H_8_N_4_, inter­molecular N—H⋯N hydrogen bonds involving the amino groups and triazole N atoms form a two-dimensional sheet.

## Related literature

For background, see: Desenko (1995 ▶); For further synthetic details, see: Van Albada *et al.* (1984 ▶). For related literature, see: Allen *et al.* (1987 ▶); Ding *et al.* (2004 ▶); Steel (2005 ▶); Van Diemen *et al.* (1991 ▶); Yi *et al.* (2004 ▶).
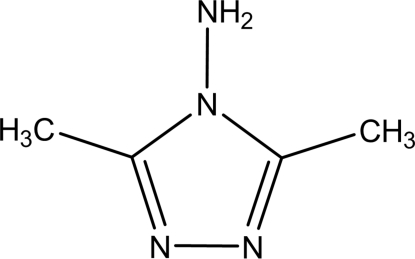


## Experimental

### 

#### Crystal data

C_4_H_8_N_4_
*M* *_r_* = 112.14
Monoclinic, 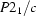

*a* = 5.8423 (12) Å
*b* = 7.7540 (16) Å
*c* = 12.846 (3) Å
β = 96.91 (3)°
*V* = 577.7 (2) Å^3^
*Z* = 4
Mo *K*α radiation
μ = 0.09 mm^−1^
*T* = 293 (2) K
0.30 × 0.30 × 0.20 mm

#### Data collection

Rigaku R-AXIS RAPID-S diffractometer
Absorption correction: none
5941 measured reflections
1333 independent reflections
1101 reflections with *I* > 2σ(*I*)
*R*_int_ = 0.031

#### Refinement

*R*[*F* ^2^ > 2σ(*F* ^2^)] = 0.051
*wR*(*F* ^2^) = 0.142
*S* = 1.12
1333 reflections
81 parameters
H atoms treated by a mixture of independent and constrained refinement
Δρ_max_ = 0.20 e Å^−3^
Δρ_min_ = −0.19 e Å^−3^

### 

Data collection: *RAPID-AUTO* (Rigaku, 1998 ▶); cell refinement: *RAPID-AUTO*; data reduction: *CrystalStructure* (Rigaku/MSC, 2002 ▶); program(s) used to solve structure: *SHELXS97* (Sheldrick, 2008 ▶); program(s) used to refine structure: *SHELXL97* (Sheldrick, 2008 ▶); molecular graphics: *SHELXTL* (Sheldrick, 2008 ▶); software used to prepare material for publication: *SHELXL97* and *PLATON* (Spek, 2003 ▶).

## Supplementary Material

Crystal structure: contains datablocks global, I. DOI: 10.1107/S1600536808014815/bv2093sup1.cif

Structure factors: contains datablocks I. DOI: 10.1107/S1600536808014815/bv2093Isup2.hkl

Additional supplementary materials:  crystallographic information; 3D view; checkCIF report

## Figures and Tables

**Table 1:** Hydrogen-bond geometry (Å, °)

*D*—H⋯*A*	*D*—H	H⋯*A*	*D*⋯*A*	*D*—H⋯*A*
N4—H4*D*⋯N1^i^	0.91 (2)	2.25 (2)	3.145 (2)	170 (2)
N)—H4*E*⋯N2^ii^	0.96 (2)	2.20 (2)	3.086 (2)	154 (2)

Symmetry codes: (i) 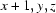; (ii) 
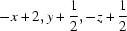
.

**Fig. 1.**
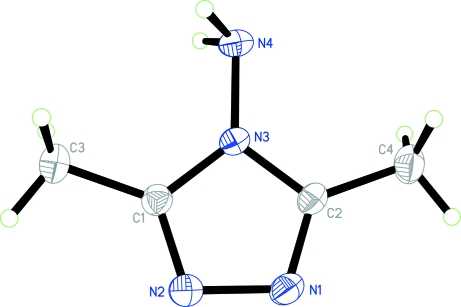
The structure of the title compound with ellipsoids drawn with 30% displacement probability.

**Fig. 2.**
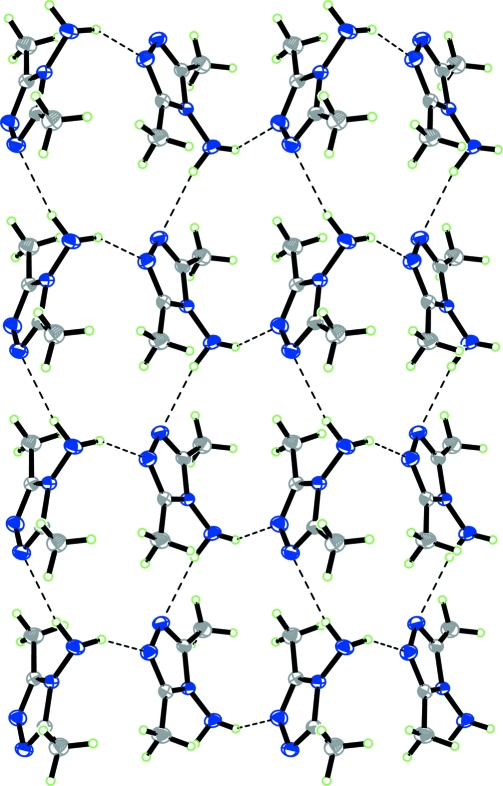
Two dimensional sheet formed by intermolecular hydrogen bonds in the title compound, with the hydrogen bonds shown as dashed lines.

## supplementary crystallographic information

### Comment

N-containing heterocyclic aromatic compounds are extensively used as bridging ligands in coordination and metallosupramolecular chemistry (Steel, 2005). For its strong σ-donor and weak π-acceptor properties, 1,2,4-triazole and its derivatives possess several coordination modes through three N donor atoms coordinating to metal ions (Van Diemen *et al.*, 1991;Yi *et al.*,2004; Ding *et al.*, 2004). We herein report the crystal structure of the title compound (I). In the molecule of (I), (Fig. 1), the bond lengths and angles are generally within normal ranges (Allen *et al.*, 1987). The H atoms of the amino group form hydrogen bonds with the N atoms of neighbouring triazole rings. The geometric parameters of the N—H···N (Spek, 2003) hydrogen-bonding interactions are given in Table 1, and a two dimensional sheet is formed by these intermolecular hydrogen bonds (Fig. 2).

### Experimental

A 80% aqueous solution of 2.6 mol of hydrazine hydrate was added slowly to 2.0 mol of acetic acid. The mixture was heated slowly and kept at 493 K for about 3 h. When the mixture was cooled, colourless block shape crystals 4-amino-3,5-dimethyl-4*H*-1,2,4-triazole were isolated.

### Refinement

Methyl H atoms were included in calculated positions and treated in the subsequent refinement as riding atoms, with C—H = 0.96Å and *U*_iso_(H) = 1.5*U*_eq_(C). Atoms H4D and H4E, which are involved in hydrogen-bonding interactions, were located in a difference Fourier map and refined freely with isotropic displacement parameters.

### Crystal data

**Table:**

C_4_H_8_N_4_	*F*_000_ = 240
*M**_r_* = 112.14	*D*_x_ = 1.289 Mg m^−^^3^
Monoclinic, *P*2_1_/*c*	Mo *K*α radiation λ = 0.71073 Å
Hall symbol: -P 2ybc	Cell parameters from 5363 reflections
*a* = 5.8423 (12) Å	θ = 3.1–27.5º
*b* = 7.7540 (16) Å	µ = 0.09 mm^−^^1^
*c* = 12.846 (3) Å	*T* = 293 (2) K
β = 96.91 (3)º	Block, colourless
*V* = 577.7 (2) Å^3^	0.30 × 0.30 × 0.20 mm
*Z* = 4	

### Data collection

**Table:**

Rigaku R-AXIS RAPID-S diffractometer	1101 reflections with *I* > 2σ(*I*)
Radiation source: fine-focus sealed tube	*R*_int_ = 0.031
Monochromator: graphite	θ_max_ = 27.5º
*T* = 293(2) K	θ_min_ = 3.1º
ω scans	*h* = −7→7
Absorption correction: none	*k* = −10→10
5941 measured reflections	*l* = −16→16
1333 independent reflections	

### Refinement

**Table:**

Refinement on *F*^2^	Secondary atom site location: difference Fourier map
Least-squares matrix: full	Hydrogen site location: inferred from neighbouring sites
*R*[*F*^2^ > 2σ(*F*^2^)] = 0.051	H atoms treated by a mixture of independent and constrained refinement
*wR*(*F*^2^) = 0.142	*w* = 1/[σ^2^(*F*_o_^2^) + (0.068*P*)^2^ + 0.1583*P*] where *P* = (*F*_o_^2^ + 2*F*_c_^2^)/3
*S* = 1.12	(Δ/σ)_max_ < 0.001
1333 reflections	Δρ_max_ = 0.20 e Å^−^^3^
81 parameters	Δρ_min_ = −0.19 e Å^−^^3^
Primary atom site location: structure-invariant direct methods	Extinction correction: none

### Special details

**Table:**

Geometry. All e.s.d.'s (except the e.s.d. in the dihedral angle between two l.s. planes) are estimated using the full covariance matrix. The cell e.s.d.'s are taken into account individually in the estimation of e.s.d.'s in distances, angles and torsion angles; correlations between e.s.d.'s in cell parameters are only used when they are defined by crystal symmetry. An approximate (isotropic) treatment of cell e.s.d.'s is used for estimating e.s.d.'s involving l.s. planes.
Refinement. Refinement of *F*^2^ against ALL reflections. The weighted *R*-factor *wR* and goodness of fit *S* are based on *F*^2^, conventional *R*-factors *R* are based on *F*, with *F* set to zero for negative *F*^2^. The threshold expression of *F*^2^ > σ(*F*^2^) is used only for calculating *R*-factors(gt) *etc*. and is not relevant to the choice of reflections for refinement. *R*-factors based on *F*^2^ are statistically about twice as large as those based on *F*, and *R*- factors based on ALL data will be even larger.

### Fractional atomic coordinates and isotropic or equivalent isotropic displacement parameters (Å^2^)

**Table:**

	*x*	*y*	*z*	*U*_iso_*/*U*_eq_	
N3	1.0528 (2)	0.17414 (16)	0.13102 (10)	0.0297 (3)	
N4	1.2366 (2)	0.2703 (2)	0.09833 (12)	0.0396 (4)	
N2	0.8357 (3)	0.02909 (19)	0.22608 (11)	0.0408 (4)	
N1	0.7072 (2)	0.06903 (19)	0.13019 (12)	0.0406 (4)	
C2	0.8418 (3)	0.1557 (2)	0.07503 (12)	0.0322 (4)	
C1	1.0418 (3)	0.0933 (2)	0.22443 (13)	0.0323 (4)	
C3	1.2408 (3)	0.0804 (3)	0.30796 (15)	0.0481 (5)	
H3A	1.1950	0.0182	0.3667	0.072*	
H3B	1.2906	0.1941	0.3299	0.072*	
H3C	1.3651	0.0206	0.2812	0.072*	
C4	0.7788 (3)	0.2255 (3)	−0.03203 (14)	0.0467 (5)	
H4A	0.6213	0.1969	−0.0559	0.070*	
H4B	0.8775	0.1762	−0.0787	0.070*	
H4C	0.7968	0.3486	−0.0308	0.070*	
H4D	1.362 (4)	0.200 (3)	0.1041 (18)	0.060 (6)*	
H4E	1.263 (4)	0.361 (3)	0.1488 (19)	0.064 (7)*	

### Atomic displacement parameters (Å^2^)

**Table:**

	*U*^11^	*U*^22^	*U*^33^	*U*^12^	*U*^13^	*U*^23^
N3	0.0243 (7)	0.0317 (7)	0.0332 (7)	−0.0002 (5)	0.0036 (5)	−0.0007 (5)
N4	0.0288 (8)	0.0462 (9)	0.0446 (9)	−0.0059 (7)	0.0082 (6)	0.0035 (7)
N2	0.0359 (8)	0.0444 (8)	0.0421 (9)	−0.0032 (6)	0.0042 (6)	0.0060 (6)
N1	0.0296 (7)	0.0453 (8)	0.0458 (9)	−0.0045 (6)	0.0003 (6)	0.0014 (7)
C2	0.0272 (8)	0.0332 (8)	0.0353 (8)	0.0019 (6)	0.0006 (6)	−0.0042 (6)
C1	0.0301 (8)	0.0323 (8)	0.0342 (9)	0.0017 (6)	0.0029 (6)	0.0009 (6)
C3	0.0413 (10)	0.0579 (11)	0.0426 (10)	0.0024 (9)	−0.0060 (8)	0.0093 (9)
C4	0.0457 (11)	0.0541 (11)	0.0378 (10)	0.0022 (9)	−0.0050 (8)	0.0020 (8)

### Geometric parameters (Å, °)

**Table:**

N3—C2	1.358 (2)	C2—C4	1.482 (2)
N3—C1	1.362 (2)	C1—C3	1.487 (2)
N3—N4	1.4123 (18)	C3—H3A	0.9600
N4—H4D	0.91 (2)	C3—H3B	0.9600
N4—H4E	0.95 (2)	C3—H3C	0.9600
N2—C1	1.305 (2)	C4—H4A	0.9600
N2—N1	1.398 (2)	C4—H4B	0.9600
N1—C2	1.306 (2)	C4—H4C	0.9600
			
C2—N3—C1	106.40 (13)	N3—C1—C3	123.42 (15)
C2—N3—N4	124.91 (14)	C1—C3—H3A	109.5
C1—N3—N4	128.54 (13)	C1—C3—H3B	109.5
N3—N4—H4D	106.9 (14)	H3A—C3—H3B	109.5
N3—N4—H4E	104.6 (13)	C1—C3—H3C	109.5
H4D—N4—H4E	109 (2)	H3A—C3—H3C	109.5
C1—N2—N1	107.45 (14)	H3B—C3—H3C	109.5
C2—N1—N2	107.31 (13)	C2—C4—H4A	109.5
N1—C2—N3	109.51 (14)	C2—C4—H4B	109.5
N1—C2—C4	126.35 (15)	H4A—C4—H4B	109.5
N3—C2—C4	124.14 (15)	C2—C4—H4C	109.5
N2—C1—N3	109.34 (14)	H4A—C4—H4C	109.5
N2—C1—C3	127.23 (16)	H4B—C4—H4C	109.5
			
C1—N2—N1—C2	−0.04 (18)	N1—N2—C1—N3	0.12 (18)
N2—N1—C2—N3	−0.05 (18)	N1—N2—C1—C3	−178.51 (17)
N2—N1—C2—C4	−179.82 (16)	C2—N3—C1—N2	−0.15 (18)
C1—N3—C2—N1	0.12 (18)	N4—N3—C1—N2	175.53 (15)
N4—N3—C2—N1	−175.76 (14)	C2—N3—C1—C3	178.54 (16)
C1—N3—C2—C4	179.90 (15)	N4—N3—C1—C3	−5.8 (3)
N4—N3—C2—C4	4.0 (2)		

### Hydrogen-bond geometry (Å, °)

**Table:**

*D*—H···*A*	*D*—H	H···*A*	*D*···*A*	*D*—H···*A*
N4—H4D···N1^i^	0.91 (2)	2.25 (2)	3.145 (2)	170 (2)
N)—H4E···N2^ii^	0.96 (2)	2.20 (2)	3.086 (2)	154 (2)

Symmetry codes: (i) *x*+1, *y*, *z*; (ii) −*x*+2, *y*+1/2, −*z*+1/2.
